# Reversed Potts Shunt as a Palliative Option for EndStage Idiopathic Pulmonary Arterial Hypertension in Childhood

**DOI:** 10.21470/1678-9741-2022-0034

**Published:** 2023-09-11

**Authors:** Livia Rocha do Valle, Cristiane Nunes Martins, Roberto Max Lopes, Fernando Antonio Fantini, Erika Correa Vrandecic, Fernando Amaral

**Affiliations:** 1 Biocor Instituto, Nova Lima, Minas Gerais, Brazil; 2 Hospital das Clínicas da Faculdade de Medicina de Ribeirão Preto, Universidade de São Paulo, Ribeirão Preto, São Paulo, Brazil

## INTRODUCTION

Idiopathic pulmonary arterial hypertension (IPAH) in children is a progressive disease with a dismal prognosis. Medical therapy is beneficial, although with a short-term effect and a five-year surviva varying from 62% to 90% between centers^[1]^. Patients afflicted with advanced symptoms demand prompt action, and new strategies have been reported. Balloon atrial septostomy creates a right-to-left shunt and decompresses the right ventricle (RV) but carries limitations as high procedural mortality, upper body oxygen desaturation, and an eventual need for reintervention^[2]^. Lung transplantation, not universally available, is an option, but median survival is around seven years and carries difficult challenges like long waiting list, donor availability, and lifelong immunosuppression^[3]^. The Potts shunt was described in 1946 as a surgical anastomosis between the descending aorta and the left pulmonary artery (PA) to allow left-to-right shunting in cyanotic congenital heart disease^[4]^. In 2004, a reversed Potts anastomosis was proposed for children with heart failure secondary to supra systemic pulmonary hypertension (PH) refractory to medical treatment^[5]^. This palliation allows reduction of PA pressure to systemic levels like what is found in children with Eisenmenger syndrome, who have a better survival than patients with IPAH^[6]^. This procedure reduces right ventricular afterload, leads to only lower body oxygen desaturation, and can serve as a bridge to transplant^[7]^. Two patients with IPAH who benefited from Potts anastomosis are presented here.

**Fig. 1:**
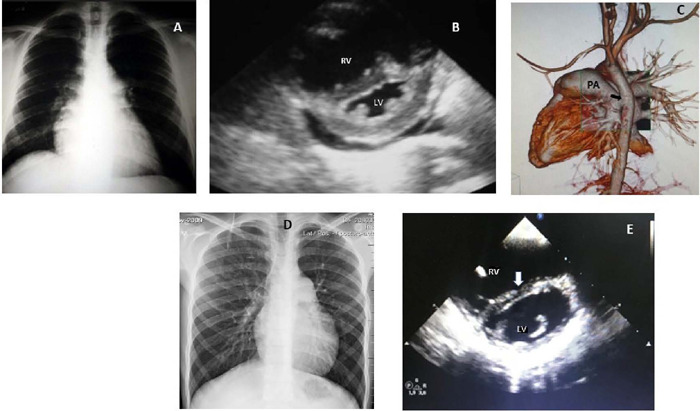
(Case Number 1) - Preoperative (A) and postoperative (D) chest radiography; preoperative (B) and postoperative (E) echocardiogram (arrow: septal curvature); (C) postoperative angiotomography (arrow: anastomosis). LV=left ventricle; PA=pulmonary artery; RV=right ventricle.

**Case Number 1:** a six-year-old boy on regular follow-up due to PAH, on bosentan 125 mg and sildenafil 60 mg daily, presented frequent syncopal episodes, New York Heart Association (NYHA) class lll-IV, O_2_ saturation 97%, and hematocrit rate 39%. The electrocardiogram (ECG) showed right bundle branch block, chest radiography showed the heart slightly enlarged, and lung flow was diminished (Figure 1 A). A small left ventricular cavity and an inverted interventricular septum (IVS) curvature suggesting increased PA pressure were seen on the echocardiogram (Figure 1B). Cardiac catheterization revealed PA pressure of 70/54/71 mmHg and increased pulmonary vascular resistance (PVR) (7.8 Wood units). In January 2015, without cardiopulmonary bypass, surgery was performed through a fourth intercostal space lateral left thoracotomy by means of applying a lateral clamp to the descending aorta on the side facing of the left PA. The left PA was clamped transversely, as were the subbranches, which were subjected to a tourniquet through a vessel-loop. Both vessels were opened longitudinally using the diameter of the descending aorta as a reference. A side-to-side anastomosis with 6-0 PROLENE® was performed between the two vessels. Thus, the diameter of the shunt was defined accordingly to the diameter of the descending aorta, and a 10-mm shunt was created. The postoperative course was uneventful, and extubation occurred 24 hours after surgery. Low adrenaline and milrinone doses plus enoxaparin were used in the first three days after operation before ward transfer. A chest angiotomography done five days after surgery showed a patent shunt (Figure 1C). He was discharged on good clinical conditions seven days after operation with a limb O_2_ saturation of 95% (upper) and 83% (lower) and a 34% hematocrit rate on the same medication. This patient was lost to follow-up until recently when an active search was successful. Reporting an active life, he was in NYHA class II with no syncopal attacks and on bosentan 125 mg/day and sildenafil 75 mg/day. Limb O_2_ saturation was 97% (upper) and 81% (lower) with no murmurs on auscultation. The heart size was normal with lung flow improvement (Figure 1D). The echocardiogram showed signs of significant PH, good right ventricular function, no IVS shift, increased left ventricular cavity, and laminar flow at the Potts anastomosis (Figure 1E) (Table 1).

**Table 1:** Echocardiographic parameters for evaluation of right ventricular function.

	Case 1		Case 2	
	Preoperative	Postoperative	Preoperative	Postoperative
PASP (mm/Hg)	121	85	125	75
PAAT (m/s)	41	100	78	110
RVET (m/s)	195	210	230	290
TAPSE (mm)	12.7	18	10.4	22
RV-PET (m/s)	118	210	95	290
FACC (%)	10	20	8.3	25

FACC=fractional area of change; PAAT=pulmonary artery acceleration time; PASP=pulmonary artery systolic pressure; RV-PET=right ventricular pre-ejection time; RVET=right ventricular ejection time;TAPSE=tricuspid annular plane systolic excursion

**Fig. 2:**
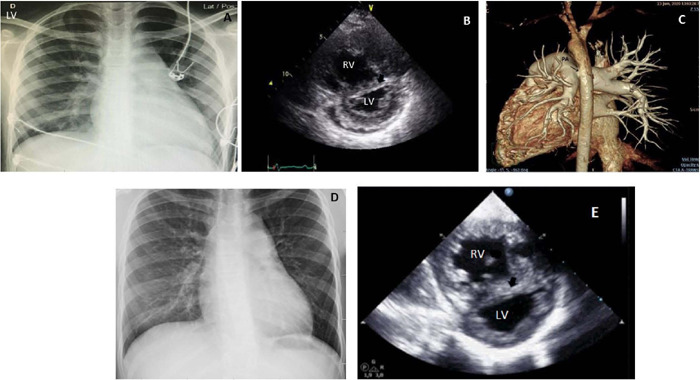
(Case Number 2) - Preoperative (A) and postoperative (D) chest radiography; preoperative (B) and postoperative (E) echocardiogram (arrow: interventricular septum); (C) postoperative angiotomography (arrow: anastomosis). LV=left ventricle; PA=pulmonary artery; RV=right ventricle.

**Case Number 2:** a 10-year-old girl on regular follow-up due tol PAH, on bosentan 125 mg/day and sildenafil 75 mg/day, was requiring recurrent hospitalizations due to frequent syncopal episodes, NYHA class IV, O_2_ saturation 95%, and hematocrit rate 44%. The ECG showed right ventricular hypertrophy and an enlarged heart was found on chest radiography (Figure 2A).The echocardiogram showed small left ventricular cavity plus IVS rectification (Figure 2B). Cardiac catheterization revealed PA pressure of 82/43/59 mmHg and increased PVR (27 Wood units). In June 2020, surgery was performed using the same technique described for Case Number 1, except that the shunt size was 7 mm. The postoperative course was uneventful, and extubation occurred 36 hours after surgery. Milrinone, dobutamine, and phenylephrine were used in the first 24 hours after operation plus blood transfusion, and ward transfer occurred three days later. She was discharged two weeks after surgery on the same medication plus furosemide 20 mg and aspirin 100 mg/day. Limb O_2_ saturation were 95% (upper) and 85% (lower) and hematocrit rate was 36%. On the day before discharge, a chest angiotomography showed a patent Potts shunt (Figure 2C). Fifteen months after surgery, she is in NYHA class II without syncope episodes on the same medication. Limb O2 saturation was 95% (upper) and 81% (lower) with no murmurs on auscultation. The heart size was normal with increased lung flow (Figure 2D) and the echocardiogram disclosed PH, improved right ventricular function, increased left ventricular cavity, and laminar flow at the Potts anastomosis (Figure 2E) (Table 1).

## QUESTIONS

**A.** Why the Potts shunt is an attractive surgical palliation for children with severely symptomatic IPAH?

**B.** Are the surgical procedure, shunt design, and postoperative management well defined?

**C.** Is there enough information regarding outcomes of patients with IPAH surgically palliated by means of a Potts shunt?

### Discussion of Questions

**Question A.** The “reversed” Potts shunt, a palliation strategy for children with end-stage IPAH, decompresses the RV and spares the coronary and cerebral circulation of deoxygenated blood^[7]^. By mimicking the Eisenmenger physiology of a patent ductus arteriosus, which has a better natural history than IPAH, the right ventricular function may be preserved and the patient may eventually undergo ung transplantation in more favorable conditions^[5,6]^.

**Question B.** The classical Potts shunt technique^[4]^ has been reported in the early experience with young symptomatic patients with severe supra systemic PH^[5,8]^. Considering the potential adverse impact of a bidirectional shunt if the PA pressure intermittently becomes subsystemic, a novel design has recently been introduced. In this approach, through a median sternotomy on cardiopulmonary bypass, a unidirectional-valved shunt is created by sewing a 12-mm Contegra™ valved conduit into a graft^[9]^. Either technique requires an experienced cardiac anesthesia team for a safe induction, and severe early postoperative complications may occur in up to 25% of the cases^[8,9]^. Considering that the patient may eventually undergo lung transplantation and that any previous approach to the chest is a relative contraindication for this type of procedure, the median sternotomy may be the preferable incisional approach for the shunt^[9]^. Stenting the ductus arteriosus is another reported option, although clinical experience is limited and debatable^[10]^.

**Question C.** Although patient selection, immediate postoperative management, and surgical technique need some refinement, the Potts shunt has been shown to be an effective palliation for these severely ill patients. After its first successful description in patients with severe PH in 2004^[5]^, promising results were reported in 2012^[11]^. Also, patients with PH secondary to other conditions were also shown to benefit from this strategy^[12,13]^. Albeit experience is restricted to a few centers, early and midterm results are available demonstrating improved functional status and longevity maximization^[7,8,9]^. Report of similar experiences by other centers as well as long-term follow-up information are needed, but this operation seems to be finding its place among the options available to assist children with such a fatal disease.

## BRIEF CONSIDERATION OF THE CASE REPORTED

These cases reflect our initial experience with Potts operation in severely ill children suffering from IPAH and should not be regarded as to represent the clinical spectrum of the patients afflicted with this disease. The preoperative and postoperative periods as well as the surgical procedure are usually demanding, and team experience with neonatal congenital heart disease is required for the success of the palliation.

## Abbreviations, Acronyms & Symbols

ECG: Electrocardiogram
FACC: Fractional area of change
IPAH: Idiopathic pulmonary arterial hypertension
IVS: Interventricular septum
LV: Left ventricle
NYHA: New York Heart Association
PA: Pulmonary artery
PA AT: Pulmonary artery acceleration time
PASP: Pulmonary artery systolic pressure
PH: Pulmonary hypertension
PVR: Pulmonary vascular resistance
RV: Right ventricle
RV-PET: Right ventricular pre-ejection time
RVET: Right ventricular ejection time
TAPSE: Tricuspid annular plane systolic excursion
